# Capsid Protein VP4 of Human Rhinovirus Induces Membrane Permeability by the Formation of a Size-Selective Multimeric Pore

**DOI:** 10.1371/journal.ppat.1004294

**Published:** 2014-08-07

**Authors:** Anusha Panjwani, Mike Strauss, Sarah Gold, Hannah Wenham, Terry Jackson, James J. Chou, David J. Rowlands, Nicola J. Stonehouse, James M. Hogle, Tobias J. Tuthill

**Affiliations:** 1 The Pirbright Institute, Pirbright, Surrey, United Kingdom; 2 School of Molecular and Cellular Biology & Astbury Centre for Structural Molecular Biology, Faculty of Biological Sciences, University of Leeds, West Yorkshire, United Kingdom; 3 Department of Biological Chemistry and Molecular Pharmacology, Harvard Medical School, Boston, Massachusetts, United States of America; Institut Pasteur, France

## Abstract

Non-enveloped viruses must deliver their viral genome across a cell membrane without the advantage of membrane fusion. The mechanisms used to achieve this remain poorly understood. Human rhinovirus, a frequent cause of the common cold, is a non-enveloped virus of the picornavirus family, which includes other significant pathogens such as poliovirus and foot-and-mouth disease virus. During picornavirus cell entry, the small myristoylated capsid protein VP4 is released from the virus, interacts with the cell membrane and is implicated in the delivery of the viral RNA genome into the cytoplasm to initiate replication. In this study, we have produced recombinant C-terminal histidine-tagged human rhinovirus VP4 and shown it can induce membrane permeability in liposome model membranes. Dextran size-exclusion studies, chemical crosslinking and electron microscopy demonstrated that VP4 forms a multimeric membrane pore, with a channel size consistent with transfer of the single-stranded RNA genome. The membrane permeability induced by recombinant VP4 was influenced by pH and was comparable to permeability induced by infectious virions. These findings present a molecular mechanism for the involvement of VP4 in cell entry and provide a model system which will facilitate exploration of VP4 as a novel antiviral target for the picornavirus family.

## Author Summary

Human rhinovirus (HRV) is a non-enveloped virus of the picornavirus family and is responsible for respiratory infections (common colds) costing billions of dollars ($) annually. There remains no vaccine or licensed drug to prevent or reduce infection. Related members of the picornavirus family include significant pathogens such as poliovirus, enterovirus 71 and foot-and-mouth disease virus, for which improved control measures are also required. A fundamental step in virus infection is the delivery of the viral genetic material through the barrier of the cellular membrane. Viruses such as HIV and influenza are enveloped in an outer membrane which can fuse with the host cell membrane to allow the viral genome to penetrate into the cytoplasm. However, non-enveloped viruses such as picornaviruses lack a membrane and the mechanisms for penetration of the membrane by these viruses remain poorly understood. The capsid protein VP4 has previously been implicated in the delivery of the picornavirus genome. In this study we demonstrate that HRV VP4 interacts with membranes to make them permeable by the formation of multimeric, size-selective membrane pores with properties consistent with the transport of viral genome through the membrane. This function of VP4 provides a novel antiviral target for this family of viruses.

## Introduction

A fundamental step in the process of viral infection is the delivery of the virus genome across the hydrophobic barrier of the host cell membrane. Enveloped viruses achieve this through the fusion of viral and target-cell membranes [1], [2]. However, non-enveloped viruses must employ alternative mechanisms to deliver their genomes into cells, such as membrane disruption or pore formation. Cell-entry studies with diverse families of non-enveloped viruses have increased our understanding of these processes and revealed common themes in the mechanisms used for membrane penetration, for example priming of virus particles by proteolytic processing and the exposure, multimerization and membrane interaction of hydrophobic or N-myristoylated viral proteins reviewed in [3], [4].

Human rhinovirus (HRV) is a small non-enveloped RNA virus belonging to the enterovirus genus of the picornavirus family, and is the most frequent cause of mild respiratory tract infections (the common cold) [5]. In recent years, it has been recognized that HRV infection is also associated with more serious clinical outcomes such as severe lower respiratory tract infections of infants and exacerbations of chronic lung diseases (asthma, cystic fibrosis and chronic obstructive pulmonary disease) reviewed in [6], [7]. Despite decades of research there is still no vaccine or licensed drug to prevent or reduce infection by HRV. The picornavirus family includes many other significant pathogens such as poliovirus (PV), enterovirus 71 (EV71), hepatitis A virus (HAV) and foot-and-mouth disease virus (FMDV), for which new or improved measures for disease control are also required.

The picornavirus particle comprises a molecule of single-stranded positive-sense RNA within a capsid of approximately 30 nm diameter. The capsid contains 60 copies of each of 4 structural proteins, VP1, VP2, VP3 and VP4, arranged with T = 1 (pseudo T = 3) icosahedral symmetry. The VP4 protein is small (predicted molecular weight of HRV VP4 approximately 7.5 kDa), hydrophobic, myristoylated at its N-terminus (post-translationally modified by the attachment of the 14-carbon saturated fatty acid, myristic acid [8]) and is located on the inside surface of the virus capsid.

Experimental data from cell culture, biochemical and structural studies have led to a proposed mechanism for entry by HRV and PV (and other closely related picornaviruses) [9]–[11] in which a ‘trigger’ that can be receptor binding and/or low pH (depending on the virus) induces major conformational changes in the virus capsid. The resulting ‘altered’ (A) particle interacts with membranes and releases the viral RNA into the cytoplasm via an undefined mechanism which is proposed to involve a channel formed in the membrane by VP4 and/or the N-terminal region of the VP1 protein. VP4 and the N-terminal region of VP1 are internal components of the mature virus but become externalised during conformational changes that result in the generation of the cell entry intermediate A particle. The A particle interacts directly with membranes without further involvement of the cellular virus receptor [12], [13]. VP1 acts to tether the particle to the membrane [12], [13] and mutagenesis of VP4 has demonstrated its involvement in both virus induced permeability in model membranes [14] and genome delivery during infection of cells [14], [15]. The interaction between virus and model membranes has recently been shown to involve an ‘umbilicus’ connecting the capsid and membrane, presumably as part of the mechanism for delivery of viral RNA across the membrane [16]. The selective release of dextrans from endosomes during infection of cells has provided evidence that membrane permeability involves formation of a pore [17] but the mechanism of pore formation remains unknown. We previously demonstrated that recombinant VP4 as a GST fusion protein was able to induce permeability in model membranes [18]. In the present study, we have produced recombinant VP4 with a minimal tag and shown that VP4 induced membrane permeability is mediated by the formation of a homo-multimeric, size-selective membrane pore.

## Results

### Recombinant VP4

**Figure 1:**
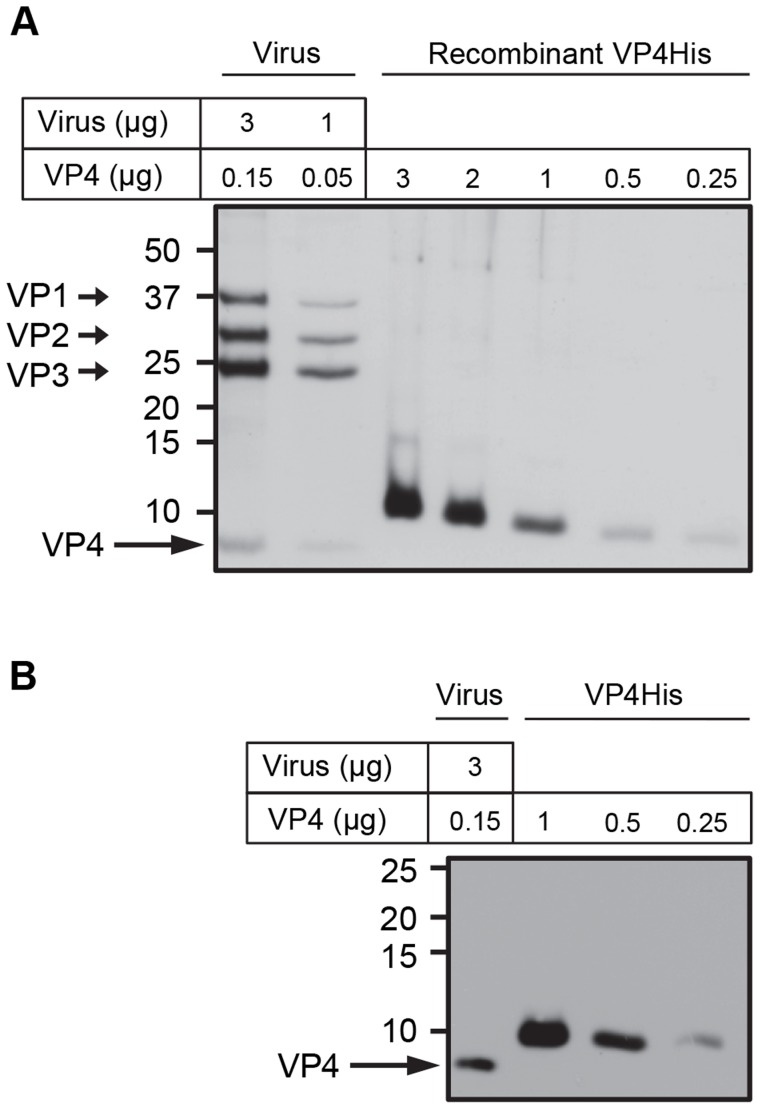
Purity and concentration of recombinant VP4 assessed by SDS-PAGE. Concentration of purified VP4His estimated by protein assay was confirmed by comparison with known quantities of native VP4 in preparations of purified virus. HRV16 (3 or 1 µg, equivalent to 0.15 or 0.05 µg VP4 respectively) and VP4His at amounts indicated, were subjected to SDS-PAGE and visualized by silver staining (**A**) or western blot using antisera to VP4 (**B**). Molecular mass markers (in kilodaltons) are indicated on the left. Arrows show expected position of the indicated viral proteins. The migration of VP4His appears slower with increasing concentration as a result of the increasing concentration of DMSO in these samples. The migration of VP4His was not altered when diluted in a constant concentration of DMSO (figure S1).

Previous studies have implicated picornavirus VP4 in viral penetration of the cell membrane and virus induced permeability in model membranes [13], [14], [19]. To further investigate the function of VP4 using biophysical approaches it was necessary to work with isolated protein. Although native VP4 can be isolated from purified virus, the amount of the protein derived from laboratory-scale virus preparations was insufficient for biophysical studies. To address this problem we previously produced recombinant HRV16 VP4 as an N-terminally myristoylated VP4GST fusion protein (VP4GST) which was able to induce membrane permeability in model membranes [18]. In the present study, the same bacterial expression system was used to produce myristoylated VP4 with a smaller tag (six-histidines at the C-terminus of VP4; VP4His). VP4His was overexpressed as insoluble inclusion bodies, purified by denaturing nickel affinity chromatography, precipitated and solubilised in DMSO, with a final yield of purified protein in the order of 0.1–0.5 mg per litre of culture. Protein purity was assessed by SDS-PAGE and silver staining and the concentration of recombinant VP4 was confirmed by comparison with native VP4 present in known quantities in purified HRV16 (Fig. 1). The migration of native VP4 and recombinant VP4His was consistent with their predicted molecular weights (approximately 7.4 kDa and 8.2 kDa respectively).

### Recombinant VP4His induces membrane permeability

**Figure 2:**
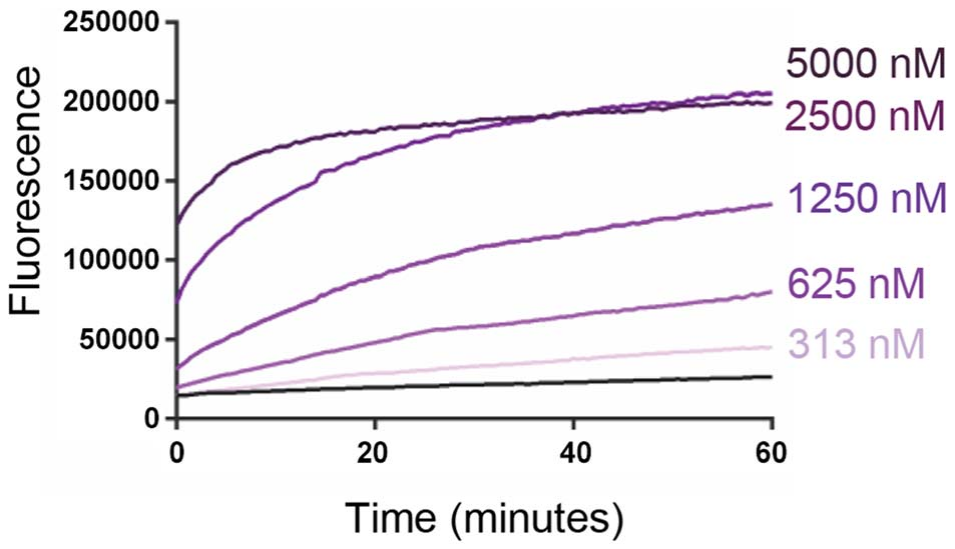
Recombinant VP4 induces dose-dependent membrane permeability. Liposomes containing carboxyfluorescein (CF) at self-quenching concentration, were mixed with VP4His at the indicated final concentrations. Membrane permeability resulting in leakage and dequenching of CF was detected by fluorescence measurements (excitation 492 nm/emission 512 nm) recorded every 30 seconds. Data shown is representative of multiple experiments (n>3). The end point fluorescent signal induced by 5000 nM VP4His was equivalent to 70–80% of the total release induced by addition of 0.5% Triton X-100.

The ability of VP4His to induce permeability in model membranes was investigated by mixing purified recombinant protein with carboxyfluorescein (CF)-containing liposomes at neutral pH and room temperature, and detecting the VP4-induced release of encapsulated dye. VP4His induced a robust signal for membrane permeability and both rate and extent of dye-release were dose-dependent (Fig. 2).

### VP4His-induced membrane permeability is myristoylation and pH dependent

For many picornaviruses, endosomal protonation triggers particle alterations involved in uncoating and membrane penetration. Different HRVs exhibit a range of sensitivities to pH mediated uncoating, depending on serotype and receptor usage. For example, uncoating of the minor group HRV2 is entirely dependent on low pH in late endosomes [20] while for the major group HRV14, receptor interactions also contribute to triggering particle alterations and this virus is therefore able to uncoat at the less acidic pH found in early endosomes [21]. In order to investigate the effect of pH on the function of VP4 we compared liposome dye-release assays at neutral pH and acidic pH values representing early (pH 6.5) and late (pH 5.5) endosomes [22].

Carboxyfluorescein (CF) fluorescence is quenched at low pH (Fig. S2) therefore the dye-release assay was first modified to allow the comparison of data collected at different pH values. End point data from dye-release assays can be expressed as a proportion of the total signal released by disruption of liposomes with detergent. End point samples can also be normalised for pH by separating the released fluorescence (by pelleting the liposomes) and adjusting to the same detergent and pH conditions as the detergent-released control. However, we wished to compare the kinetics of dye-release (not just end points) and therefore required an alternative procedure for normalising real-time CF dye-release assays carried out at different pH values.

**Figure 3:**
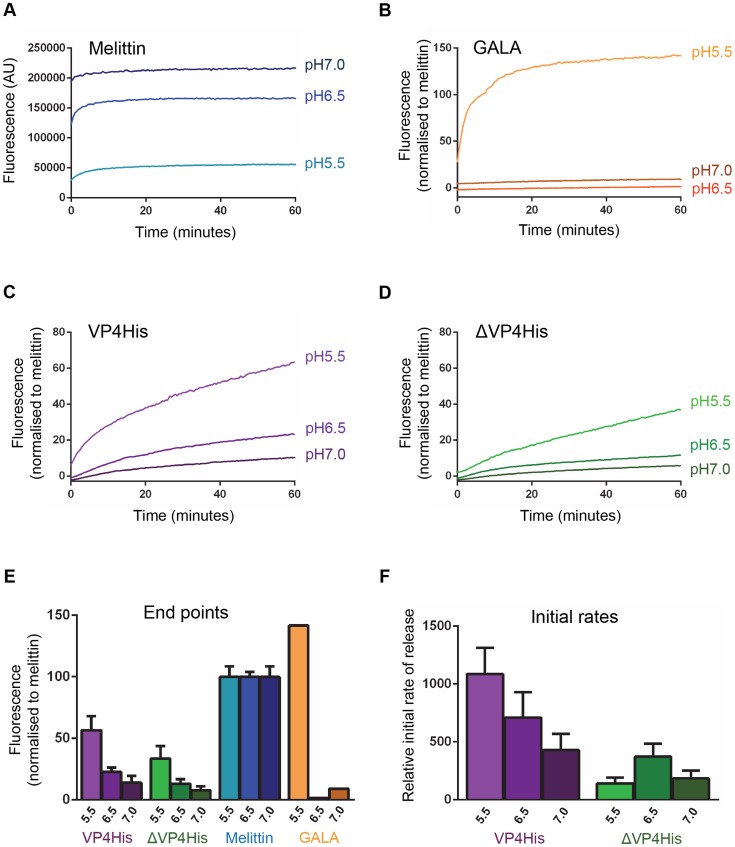
VP4-induced membrane permeability is enhanced by low pH and VP4 myristoylation. Carboxyfluorescein (CF)-containing liposomes at pH 7.0, pH 6.5, and pH 5.5, were mixed with the following peptides or proteins and membrane permeability resulting in leakage and dequenching of CF was detected by fluorescence measurements (excitation 492 nm/emission 512 nm) recorded every 30 seconds. **A**) The pH-independent pore-forming peptide melittin at 10 µM. AU, arbitrary units. CF fluorescence is known to be less efficient at low pH and the apparent reduction in melittin-induced signal at low pH values (pH 6.5 and pH 5.5) could therefore be restored to the same level as the pH 7 sample by adjusting released CF in supernatants to neutral pH (data not shown), thus confirming that melittin-induced permeability was unaffected by pH and demonstrating a requirement to compensate for the reduced efficiency of CF fluorescence at low pH. Therefore, data in the remaining panels (B–F) was normalized to the maximum signal induced by melittin at each pH value. **B**) the acid-dependent pore-forming peptide GALA at 1 µM. **C**) VP4His at 5 µM. **D**) ΔVP4His (unmyristoylated) at 5 µM. **E**) End point and **F**) initial rates are shown to summarise the data in panels A–D. Data shown is representative of multiple experiments (n>3). Error bars represent standard error of the mean of values from 3 experiments.

We therefore used the well-characterised pH independent pore-forming peptide melittin [23], [24] as a control for the liposome dye-release assay. The permeability induced by melittin appeared reduced at low pH due to the quenching of CF fluorescence at low pH (Fig. 3A). However, when samples were separated from liposomes and adjusted to neutral pH as described above, the signal in all samples became equivalent to the pH 7.0 sample. This confirmed that melittin induced permeability was indeed pH independent and that dye-release data generated at a given pH could be expressed as a proportion of the maximum release by a melittin control at that same pH. This provided a method for normalisation of data which corrected for the low-pH quenching of CF and allowed real-time data generated at different pH values to be directly compared.

The pore-forming peptide GALA, which only forms pores in model membranes at pH<6 [25], [26] was used as an additional control. As expected GALA did not induce permeability at pH 7.0 or pH 6.5 but induced robust permeability at pH 5.5 (Fig. 3B).

Native VP4 is myristoylated at the N-terminus. The effect of the myristate group on the induction of membrane permeability by VP4His was therefore determined by comparing myristoylated (VP4His) and unmyristoylated (ΔVP4His) forms of recombinant protein in the liposome dye-release assay. Both forms of VP4 induced membrane permeability but VP4His (myristoylated) generally induced more extensive membrane permeability with a higher initial rate of release than ΔVP4His (unmyristoylated) (Fig. 3C & D). The permeability induced by both VP4His and ΔVP4His increased at more acidic pH values (Fig. 3C, D, E). However, VP4His consistently induced more extensive permeability than ΔVP4His at all pH values tested (Fig. 3E). Analysis of the dye-release kinetics showed that the initial rate of release induced by VP4His increased dramatically with decreasing pH, but the corresponding effect was not seen with ΔVP4His (Fig. 3F).

No significant permeability was seen in control samples exposed to low pH conditions in the absence of protein/peptide. Together these findings suggest that VP4 membrane interactions are more efficient at low pH and that the presence of myristate is required for maximum membrane activity of VP4 under the physiological conditions encountered during HRV entry.

### VP4-induced membrane permeability is size-selective

**Figure 4:**
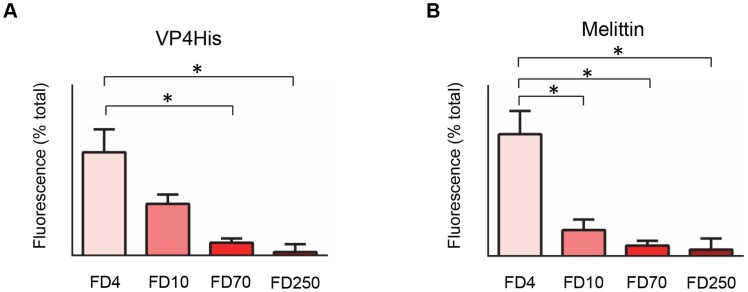
VP4-induced permeability is size-selective. Liposomes containing FITC-labelled dextrans of 4 kD (FD4), 10 kD (FD10), 70 kD (FD70) or 250 kD (FD250) were mixed with 5 µM VP4His (**A**) or 10 µM melittin (**B**). Release of dextrans was quantified by pelleting the liposomes and measuring the fluorescence in the supernatant. Data is presented as percentage of total release observed by lysis of liposomes by addition of detergent. Error bars represent standard error of the mean (n = 3) and asterisks indicate statistical significance calculated by one way Anova (p*<0.05). Data is representative of multiple independent experiments.

We hypothesised that VP4-induced membrane permeability was the result of formation of discrete pores with dimensions sufficient for transfer of macro-molecules such as RNA across the membrane. This was investigated by measuring the VP4-induced release from liposomes of FITC-labelled dextrans of different sizes. Smaller dextrans of 4 kDa (Stokes' radius of 14 Å) and 10 kDa (Stokes' radius of 23 Å) were indeed released from liposomes with higher efficiency than 70 kDa (Stokes' radius of 60 Å) and 250 kDa (Fig. 4A). The pore forming toxin melittin was included as a control for these studies and released only the smallest dextran tested (Fig. 4B), in agreement with previous reports [24], [27]. These experiments indicate that VP4 permeability is via formation of size-selective membrane pores which limit the transfer of molecules with Stokes' radius approaching ∼60 Å. Such a pore, with a lumen diameter ≤12 nm would be consistent with the size required for passage of single-stranded nucleic acid.

### VP4 multimerizes to form a pore structure

**Figure 5:**
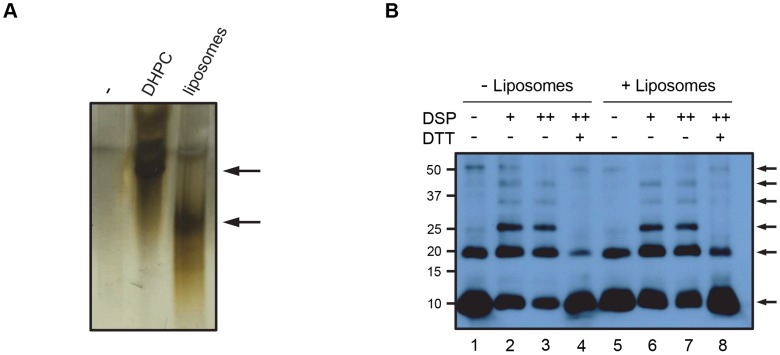
VP4 forms a multimeric complex. **A.** VP4His was incubated in the presence of the membrane-mimetic detergent DHPC or liposomes, or mock-treated (−), resolved by native PAGE and visualised by silver staining. The position of bands shown by arrows indicates potential migration of VP4His into the gel. **B**. VP4 was incubated with (+) or without (−) liposomes and crosslinked by the addition of DSP at 0.5 mM (+) or 1 mM (++). Samples were resolved by non-reducing SDS-PAGE and detected by western blot using antisera to VP4. DTT was used in some samples to reverse the crosslinking prior to SDS-PAGE. Arrows indicate VP4 multimers. Molecular mass markers (in kilodaltons) are indicated on the left.

Membrane pores formed by small pore-forming proteins or peptides often have a homo-oligomeric structure. Multimerization seemed the likely mechanism for VP4 pore formation. We therefore investigated the ability of VP4 to form a homomultimeric complex by analysing samples by PAGE. Purified VP4His was difficult to detect when analysed directly by native PAGE (non-denaturing conditions) and silver staining (Fig. 5A) perhaps due to solubility or charge which did not permit efficient electrophoresis. However, after mixing with liposome membranes or the membrane mimetic detergent DHPC, VP4 appeared to be readily detected (Fig. 5A), suggesting that interaction with micelles or multimerization was able to facilitate migration into the gel.

In contrast to the results with native PAGE, VP4His was readily detected by SDS-PAGE (see Fig. 1). Samples of VP4His in the presence of liposomes were therefore treated with the amine-reactive chemical cross-linker dithiobis (succinimidyl propionate) (DSP), separated by SDS-PAGE and identified by western blot. Crosslinking in the presence of liposomes resulted in detection of a characteristic ladder of bands, corresponding in size to monomeric VP4His (predicted size of approximately 8.2 kDa) and cross-linked multimers of VP4His in increasing numbers, up to a hexameric complex migrating with apparent molecular weight of approximately 50 kDa (Fig. 5B, lanes 6 and 7). The presence of species with uniform and incremental increases in apparent molecular weight equivalent to the addition of VP4His monomers, suggested multimerization of monomeric species rather than random aggregation. Upon reversing the crosslinking with DTT, the presence of multimers was diminished and an increase in monomer was observed (Fig. 5B, lane 8), implying that the multimeric units were indeed comprised of VP4His monomers. Interestingly, VP4His multimerization was also detected in the absence of crosslinking; in these samples a band equivalent to the dimeric and hexameric complexes were clearly seen but intermediate multimers were of reduced intensity (Fig. 5B, lane 5), similar to the appearance of samples after reversal of crosslinking. This suggested that the putative hexameric structure was more stable than the intermediates, and could survive the denaturing conditions of SDS-PAGE, reinforcing the case for this structure as a biologically relevant assembly. VP4His multimerization was also detectable in the absence of membranes (Fig. 5B, lanes 1–4), suggesting that multimerization may be an intrinsic property of the protein which is membrane-independent. Alternatively, the detergent used in preparation for SDS-PAGE may have provided a micellar environment which promoted multimerization. VP4 multimerization was not observed after disrupting virus with SDS in preparation for SDS-PAGE (see Fig. 1), however, the amount of VP4 in virus-derived samples was relatively low such that multimers may be more difficult to detect.

### VP4 multimeric structure visualized by electron microscopy

**Figure 6:**
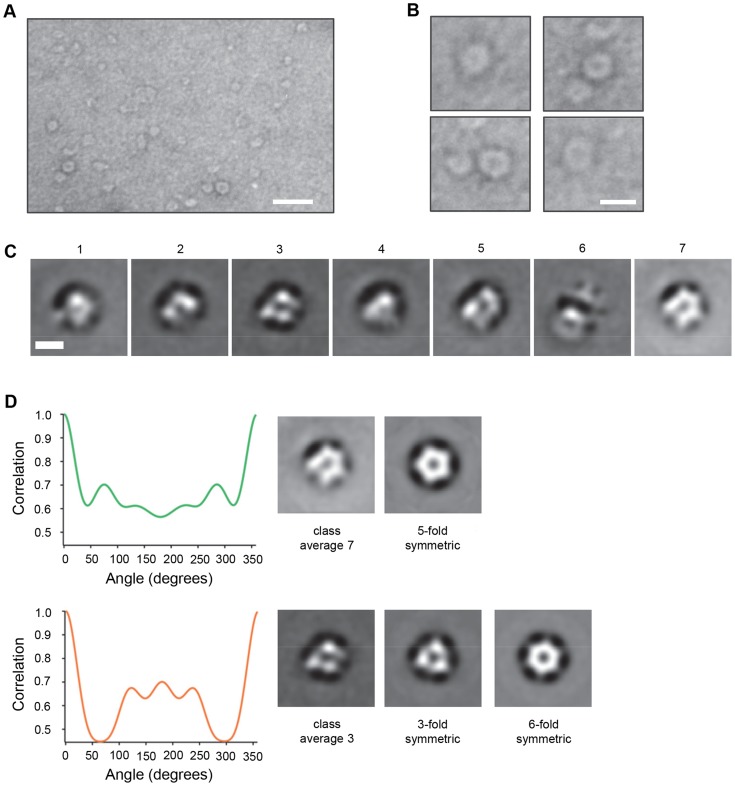
VP4 multimeric structure visualized by electron microscopy. **A.** VP4GST was reconstituted in the membrane mimetic detergent DPC, applied to carbon coated grids and stained with uranyl formate. Scale bar = 20 nm. **B**. Individual ring-like structures were manually selected and cropped from unprocessed digital images. Scale bar = 10 nm. **C**. Class average images of particles automatically picked from a large image data set and classified into 7 classes. Scale bar = 5 nm. **D**. Class averages 3 and 7 (top-down views) analyzed by rotational harmonic analysis.

To facilitate detection of multimeric VP4 complexes by electron microscopy, we used recombinant VP4 fused to a larger tag, the 26 kDa glutathione *S*-transferase (VP4GST). This protein was previously shown to be functional for interaction with and induction of permeability in model membranes [18]. In the study reported here, myristoylated VP4GST was mixed with the membrane mimetic zwitterionic detergent dodecylphosphocholine (DPC), protein-micelle complexes were purified by size exclusion chromatography and examined by negative stain transmission electron microscopy. Ring-like structures were readily observed (Fig. 6a and 6b) consistent with ‘top-down’ views of multimeric pores. We also initially tried to image VP4 in liposomes but in our hands the negative stain images were of poor quality while reconstitution of VP4 in detergent gave far superior results. We therefore took forward the latter strategy to generate the data shown in the manuscript. An image data set representing all detected structures (n = 5929) was separated into seven class averages (Fig. 6c). Several different classification schemes were tested with varying numbers of classes, and a K-means classification with seven classes gave the most consistent and interpretable classes. We believe the overall micelle-pore complexes (VP4GST+micelle) would in fact be wider than they are high, therefore forming an overall disc shape which would preferentially lie flat on the grid perhaps favouring orientation of the pore facing upwards and reducing the number of alternate views. Of the seven classes, two were selected (class 3 and 7 in Fig. 6c) as clear top-down views (the remainder appeared to represent mis-alignments or side views of the complex) and analysed by rotational harmonic analysis which indicated 5- or 6-fold symmetry (Fig. 6d), consistent with a pentameric or hexameric complex of VP4GST molecules. The initial appearance of the class 3 average as a complex with 3-fold symmetry may be due to the propensity of the GST fusion partner to form dimers, thus initially presenting six GST moieties as three dimers. However, the appearance of the class 7 average as a pentameric structure suggested that GST dimerization was not controlling the stoichiometry of the complex. It is conceivable that recombinant VP4 is able to form pore structures with different numbers of multimers; perhaps a single preferred structure for the native virus-derived pore may be influenced by additional factors.

### VP4-induced membrane permeability is comparable to that of virus

**Figure 7:**
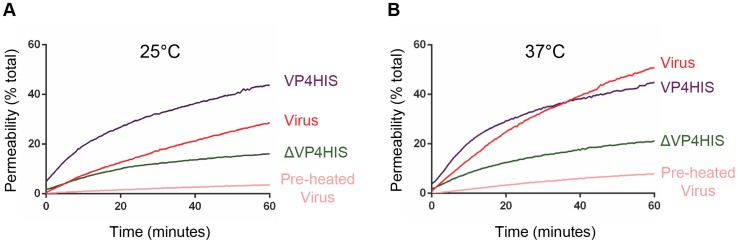
VP4-induced permeability is comparable to that of virus. Carboxyfluorescein-containing liposomes were mixed with VP4His at 5 µM (equivalent to approximately 5 µg/assay) or 1 µg HRV16 (equivalent to 50 ng VP4/assay) and membrane permeability detected by fluorescence measurements recorded every 30 seconds. Assays were conducted at 25°C (**A**) or 37°C (**B**). Only a minority proportion of recombinant protein is thought to take part in the reaction. Data is presented as % of total end-point release observed by lysis of liposomes by addition of detergent. Data shown is representative of multiple experiments (n>3).

We wished to confirm that the function of recombinant VP4 was relevant to the membrane interactions of virus particles. Previous studies demonstrated that PV was able to induce electrical conductance channels in planar model membranes [19], [28]. We therefore compared the membrane permeability induced by VP4His and purified HRV16, by measuring CF leakage from liposomes in the dye-release assay. Recombinant VP4 and purified virus induced membrane permeability with broadly similar characteristics (Fig. 7). However, virus-induced permeability was more efficient at 37°C than at 25°C, while the recombinant VP4-induced permeability was relatively unaffected by the change in temperature. These observations demonstrate a temperature dependence for HRV-membrane interactions, consistent with previous studies with PV [19] and with a model of temperature dependent particle breathing which controls the exposure and membrane interactions of internal capsid components such as VP4 [29], [30]. Although studies with PV demonstrated breathing only at physiological temperatures approaching 37°C [29], studies with HRV have shown particle breathing at lower temperatures such as 25°C [30]. Importantly, virus samples pre-treated at 60°C to convert virions into empty particles induced only low levels of permeability, most likely because empty particles would no longer contain VP4.

**Figure 8:**
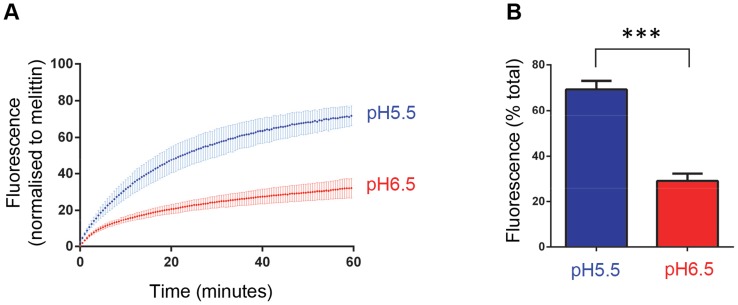
HRV-induced membrane permeability is enhanced by low pH. **A**) Carboxyfluorescein (CF)-containing liposomes at pH 6.5 and pH 5.5 were mixed with HRV (0.5 µg) and membrane permeability resulting in leakage and dequenching of CF was detected by fluorescence measurements (excitation 492 nm/emission 512 nm) recorded every 30 seconds (as in figure 3). **B**) End points of HRV induced membrane permeability. Data in panels A and B was normalized to the maximum signal induced by melittin at each pH value. Data shown is representative of multiple independent experiments (n>3). Error bars represent standard error of the mean (n = 3) and asterisks indicate statistical significance calculated by one way Anova (p*<0.05).

**Figure 9:**
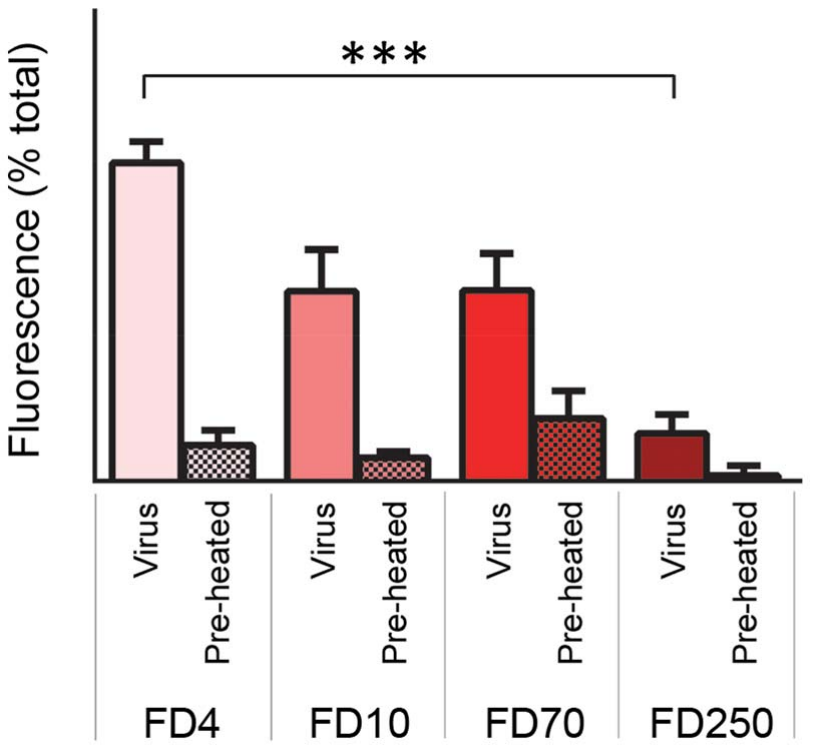
HRV-induced permeability is size-selective. Liposomes containing FITC-labelled dextrans of 4 kD (FD4), 10 kD (FD10), 70 kD (FD70) or 250 kD (FD250) were mixed with 0.5 µg HRV or preheated HRV. Release of dextrans was quantified by pelleting the liposomes and measuring the fluorescence in the supernatant (as in figure 4). Data is presented as percentage of total release observed by lysis of liposomes by addition of detergent. Error bars represent standard error of the mean (n = 3) and asterisks indicate statistical significance calculated by one way Anova (p*<0.05). Data is representative of three independent experiments.

The membrane permeability induced by recombinant VP4 was enhanced at low pH (Fig. 3). We therefore investigated the effect of pH on virus-induced permeability by comparing the permeability induced by virus at pH 6.5 (representing early endosomes) and pH 5.5 (representing late endosomes) (Fig. 8). This demonstrated that virus-induced permeability was significantly enhanced at pH 5.5, consistent with this lower pH contributing to the triggering of particle alterations involved in membrane interactions and uncoating. In addition, we carried out dextran release studies to investigate if virus-induced permeability was size-selective, as shown earlier for recombinant VP4 (Fig. 4). Virus-induced permeability was indeed also size-selective, with the smaller dextrans being released to a significantly greater extent than the larger dextran (Fig. 9). This confirmed that virus-induced permeability was not via the large scale disruption of the membrane but was also likely to involve a defined pore. However, while the results for the VP4 pore suggested limiting the transfer of molecules with Stokes' radius approaching ∼60 Å, the virus-induced permeability permitted partial release of these molecules, appearing to have a larger size cut-off, perhaps consistent with the involvement of additional capsid components in the virus-induced pore such as the N-terminus of VP1.

## Discussion

Previous studies have demonstrated that during picornavirus cell entry, the internal capsid protein VP4 is released from the virus and interacts with cell membranes. Furthermore, it has been shown that mutations in VP4 alter both the ability of the virus to induce permeability in model membranes and to deliver the viral genome into the cell [14]. In the present study we have characterised the ability of recombinant HRV VP4 to induce permeability in model membranes and demonstrated that VP4 forms a multimeric, size-selective membrane pore, with dimensions consistent with the transport of viral RNA.

Native VP4 is myristoylated at its N-terminus. This modification is often a feature of proteins with membrane binding properties [31], [32]. Existing mutagenesis studies have implicated VP4 as important for the formation of pores in electrophysiology experiments and for RNA release [14], and the addition of the N-terminal nine amino acids (including myristoylation signal) of PV VP4 to GFP was previously shown to target the fusion protein to membranes [33]. Myristoylation of VP4 has been shown to be essential for the assembly of infectious virions, unfortunately making it difficult to further probe the role of myristoylation of VP4 in pore formation in the context of the intact virus. Although myristoylation was not essential for the formation of functional pores by recombinant VP4, our data show that it significantly improves pore formation and probably acts by increasing the efficiency of initial membrane interaction.

Conditions of low pH appeared to favour both VP4 and virus induced permeability, increasing the extent and rate of permeability, suggesting that membrane activity may be optimised for the low pH conditions encountered in acidified endosomes, the site of membrane penetration by HRV. Permeability was greater at pH 5.5 (the pH of late endosomes) relative to pH 6.5 (the pH of early endosomes) which is in contrast to the early endosome as the site of uncoating of the major group HRV14 [21]. The site of uncoating for HRV16 (from which the recombinant VP4 was derived) has not been determined. However, HRV16 interacts less efficiently than HRV14 with the major group receptor ICAM-1 [34] and requires a lower pH to trigger uncoating *in vitro* [35], suggesting that uncoating of HRV16 could indeed involve a cellular environment closer to that of the late endosome and consistent with our data for optimal membrane permeability at pH 5.5. The effect of low pH was most pronounced for myristoylated VP4, again suggesting that myristoylation is required for maximal activity. In these studies, low pH may act to promote VP4-membrane interaction, multimerization, solubility or pore stability. However, not all picornaviruses penetrate the membrane from within acidified vesicles and the increase in pore forming ability at lower pH seen with HRV16 VP4 may not apply universally across the virus family.

The permeability induced by recombinant VP4 was size-selective, such that dextrans of 4 and 10 kDa were released from liposomes efficiently, relative to larger dextrans of 70 and 250 kDa. Permeability induced by virus was also size-selective but allowed partial release of the intermediate size dextrans (10 and 70 kDa), perhaps suggesting virus derived pores are larger, consistent with the potential involvement of additional capsid components such as the N-terminus of VP1. Together this strongly suggested that release of dextrans was via a pore in the membrane and that permeability did not involve large scale membrane disruption or lysis of liposomes. This confirmed an earlier study in which liposomes permeabilized by recombinant VP4 remained intact [18] and is consistent with ion channel formation by PV [28], the size-selective release of dextrans from endosomes during infection of cells by the minor group HRV2 [17] and the transfer of genome into intact liposomes by both HRV2 [36] and PV (unpublished data). An alternative model for major group HRV14 membrane penetration has been proposed to involve endosome disruption instead of pore formation reviewed in [11]. Although our current findings with recombinant VP4 of HRV16 are not consistent with membrane disruption, both models may be possible due to subtle differences between viruses in terms of pH sensitivities, involvement of receptor and proportion of VP4 released.

Recombinant VP4 was only visualized by native PAGE after mixing with liposomes or detergent (Fig. 4), suggesting that VP4 was altered, or formed a complex, with the micellar environment provided by these reagents. Based on the majority of evidence indicating a model of pore formation by picornaviruses, we hypothesised that VP4 permeability probably involved formation of a discrete pore structure, which for a protein the size of VP4, would only be possible by formation of a multimeric complex. Chemical cross-linking and SDS-PAGE confirmed the ability of recombinant VP4 to multimerize and suggested the formation of a stable hexameric complex. VP4 complexes visualised by TEM confirmed the formation of a multimeric pore and harmonic analysis suggested a pentameric or hexameric structure. For many years the picornavirus virion was predicted to interact with the membrane and release its RNA at a capsid vertex at the 5-fold symmetry axis. Furthermore, receptor binding was shown to align the PV particle with a five-fold axis of symmetry pointing towards the membrane [37]. It was therefore tempting to speculate that 5 copies of externalised VP4 would simultaneously insert into the membrane and form a pentameric complex. However, both PV and HRV2 are thought to disengage with their receptors before membrane penetration occurs, so that an alternative alignment with the membrane (away from the 5-fold) was also possible. In recent years it has become clear that indeed neither the site of RNA release or site of externalisation of capsid components is at the five-fold axis [38]–[41]. A recent study has visualized PV linked to liposome membranes by a long connector or umbilicus during the act of RNA translocation [16]. The virus is tilted away from the 5-fold to expose the quasi-3-fold axis (the site of externalisation of VP1) to the membrane. The connector is likely to contain capsid protein VP1 (the capsid footprint of the connector coincides with the VP1 exit site). How the putative multimeric complex formed by recombinant VP4 is related to this structure is unknown, perhaps the VP4 structure provides the membrane pore, while the externalised VP1 molecules provide the connector.

Chemical crosslinking revealed that VP4 forms a multimeric complex even in the absence of membranes, this could be evidence that VP4 multimerizes prior to membrane insertion. This possibility is supported by a previous study which demonstrated that two copies of VP4 could be cross-linked together on the surface of HRV particles during the transient externalisation of capsid components that occurs in particle ‘breathing’ [42]. In the current study VP4 dimers were also observed as the predominant form of multimer. In an alternative model where membranes are required for VP4 multimerization, it is possible that detergent micelles encountered in preparation for SDS-PAGE were able to provide a membrane-like environment which promoted VP4 multimerization. Although perhaps counter-intuitive that such a multimer would survive boiling in SDS, similar highly stable multimers have been reported for other small hydrophobic viral pore forming proteins such as the hepatitis C virus p7 protein [43].

The release of fluorescent dyes from liposomes by recombinant VP4His appeared to share similarities to that induced by virus. The comparison of directly equivalent amounts of VP4His and virus particles was difficult due to the expectation that the very hydrophobic nature of VP4His would cause it to have low solubility when diluted into the assay, making it likely that only a minor proportion of the recombinant protein would take part in the membrane interaction. However, we speculated that if the active proportion of VP4His was only 1% of the total (5 µg), this would be equivalent to 50 ng of active VP4His per assay, approximately equivalent to the amount of native VP4 provided by 1 µg of virus. Virus induced permeability was more temperature sensitive, but this could be entirely consistent with virus-membrane interactions and induction of permeability being related to particle ‘breathing’ which are increased at physiological temperatures. The similarities between the VP4- and virus–induced permeability could be interpreted as suggesting that VP4 is solely responsible for membrane permeability during virus entry. However, the finding that virus-induced permeability allowed release of larger dextran molecules relative to VP4His, suggests that the virus-derived pore might be larger, consistent with the potential involvement of additional capsid components in the pore, such as the N-terminus of VP1.

The size-selective pore formed by recombinant VP4, which permits transfer of dextrans of 4 kDa (Stokes' radius of 14 Å) and 10 kDa (Stokes' radius of 23 Å) but restricts dextrans of 70 kDa (Stokes' radius of 60 Å) or above would suggest a pore with lumen diameter of between approximately 4.6 nm and 12 nm. Multimeric VP4 pore complexes visualized by TEM were consistent with this size. For the virus derived pore, which restricts the passage of dextrans of 250 kD (Stokes' radius of 105 Å) the lumen diameter would be predicted to have an approximate dimension of between 12 nm and 21 nm. These potential channel dimensions can only be considered as approximate estimates. However, since the potential role of the pore is transfer of the viral genome, we were encouraged to consider the dimensions of existing channels capable of transporting RNA. Structural models of cellular RNA polymerases include an approximately 30 Å long, 15 Å wide channel, known as the “RNA-exit-channel,” through which the nascent single strand of RNA exits the polymerase [44], [45]. Alpha-hemolysin is a bacterial heptameric transmembrane pore through which single stranded molecules of DNA or RNA can be driven by an applied electric field. During this process, nucleotides can be detected passing through the channel pore in sequential order due to the limiting diameter of the pore, which at its narrowest point is 14 Å in diameter [46]. Therefore a channel 14–15 Å in diameter could be considered the absolute minimal size required for active transport of unstructured single stranded RNA. In the case of genome delivery by a picornavirus our findings suggest the involvement of a much larger pore, perhaps not surprising considering both the lack of energy for active transport of a long molecule through a constricted channel and the potential for secondary structure in the genomic RNA.

In recent years, entry inhibitors have been developed that prevent exposure, multimerization or membrane insertion of the viral fusion proteins of enveloped viruses such as dengue virus and HIV [47], [48] and as reviewed in [49], [50]. For picornaviruses, the exposure, multimerization and membrane insertion of VP4 appears to be the equivalent process. The findings in this study present the first direct evidence of multimerization and pore formation by picornavirus VP4. This provides a novel molecular mechanism for VP4-mediated membrane permeability during picornavirus entry and suggests that VP4 be considered as an antiviral target for this family of viruses.

## Materials and Methods

### Construction of VP4 expression plasmid

The sequence encoding HRV16 VP4 was amplified from pET23d VP4GST [18] by PCR using primers VP4F (5′-TATACC**ATG**GGCGCTCAAGTATCTAGACAGAATG-3′) and VP4HisR (5′-TATACTCGAG **TCA**ATGGTGATGGTGATGGTGTTGCAGAGTG-3′). The resulting sequence encoded VP4 with an additional six histidines at the C-terminus and flanked by start and stop codons (indicated in bold) and restriction sites (underlined). This DNA was cloned into pET23d (Novagen) to create pETVP4His. Insert integrity was confirmed by sequencing.

### Expression and purification of recombinant VP4

Recombinant VP4 was expressed in *E. coli* (BL21 DE3 pLysS) as myristoylated protein (VP4His) by co-transformation of bacteria with pETVP4His and a plasmid encoding *N*-myristyltransferase (pET-NMT), or as unmyristoylated protein (ΔVP4His) by transformation with only pETVP4His. The myristoylation of recombinant VP4 in this system was previously confirmed by mass spectrometry [18]. Expression was induced by the addition of 0.2 mM isopropyl β-D-1-thiogalactopyranoside (IPTG). Myristic acid was added to a final concentration of 5 µg/ml to cultures co-transformed with pET-NMT. Bacteria were lysed in denaturing lysis buffer (7M urea, 1% Triton X-100, 20 mM HEPES pH 7.5), sonicated and the clarified supernatant applied to a pre-packed nickel affinity column (GE Healthcare). Proteins were eluted with increasing concentrations of imidazole and fractions analysed by SDS-PAGE and Coomassie staining or western blot. Fractions containing VP4 were pooled and dialysed sequentially against decreasing concentrations of urea after which precipitated VP4His was collected by centrifugation and resuspended in DMSO. Recombinant myristoylated VP4 tagged with GST (VP4GST) was similarly expressed using pET23d-VP4GST and pET-NMT [18] and purified as described above for VP4His.

### Purification of virus

HRV16 derived from an infectious cDNA plasmid [51] was propagated in HeLa Ohio cells and purified using standard methods [52]. Briefly, infected cell cultures were lysed by freeze/thawing and clarified supernatants concentrated by precipitation with ammonium sulphate. Virus particles were purified by pelleting through a cushion of 20% (w/v) sucrose in PBS followed by sedimentation in a sucrose density gradient comprising 15–45% (w/v) sucrose in PBS. Purified virus was quantified by absorbance at 260 nm, where an optical density of 7.7 = 1 mg/ml. For some membrane permeability experiments virus was preheated at 60°C for 10 min to convert native virus into empty particles.

### Preparation of liposomes

Liposomes comprising of phosphatidic acid, phospatidylcholine, cholesterol and rhodamine-labelled phosphatidylethanolamine (Avanti Polar Lipids) (molar ratios 44.5∶44.5∶10∶1 respectively) were prepared as previously described [13] by rehydration of dried lipid films in 107 mM NaCl, 10 mM Hepes pH 7.5 and extrusion through 400 nm pore-size membranes using a mini-extruder (Avanti Polar Lipids). The lipid concentration of liposome preparations was estimated by comparing the level of rhodamine fluorescence in the liposome sample relative to samples of rehydrated lipids of known concentration. The expected diameter (average 400 nm) and size distribution of liposomes was confirmed by dynamic light scattering (Malvern Zetasizer µV). Liposomes containing carboxy-fluorescein (CF) (Sigma) were prepared by rehydrating lipids in the presence of 50 mM CF, 10 mM Hepes pH 7.5. Liposomes containing FITC-conjugated dextrans (FD; Sigma) were prepared by rehydrating lipids in the presence of 25 mg/ml FD, 107 mM NaCl, 10 mM Hepes pH 7.5. Liposomes containing CF or FD were purified from external fluorescence by multiple cycles of ultracentrifugation [18] and resuspended in107 mM NaCl, 10 mM Hepes pH 7.5.

### Membrane permeability assays

Membrane permeability was measured by detecting the release of fluorescent material from within liposomes. Purified liposomes containing CF or FD were added to test substances (recombinant protein, peptide, virus or mock controls in typical volume 5 µl) to give typical final concentrations of 50 µM lipid,107 mM NaCl, 10 mM HEPES pH 7.4 and total volume of 100 µl. Reagents and plastic-ware were pre-equilibrated to the reaction temperature (25°C or 37°C).

For CF release, reactions were assembled in 96-well plates and membrane permeability detected in real time by the release, dequenching and increase in fluorescence of CF. Measurements were recorded every 30 s for 1 hr using a fluorescence plate reader with excitation and emission wavelengths of 485 nm and 520 nm respectively (Plate CHAMELEON V, Hidex). Initial rates were calculated from the linear slope of lines generated from the initial four data points.

For FD release, permeability reactions were incubated for 1 hr, liposomes pelleted at 100,000× g for 30 mins and fluorescent signal in the supernatant measured using the plate reader as above. The signal released by the addition of 0.5% (v/v) Triton X-100 was taken as 100% and all supernatants were adjusted to 0.5% (v/v) Triton X-100 prior to recording fluorescent measurements, to normalise for fluorescence quenching by presence of detergent.

For experiments investigating the effect of pH on the induction of membrane permeability, CF release reactions were assembled with 10 mM sodium citrate/citric acid buffer at pH 5.5, 6.5 or 7.0 (instead of HEPES). CF fluorescence is quenched at low pH. Experiments to confirm this were carried out by adjusting a stock solution of CF (50 mM CF, 10 mM Hepes pH 7.5), to different pH values by 100-fold dilution in the citrate/citric buffers described above. After 1 hour, samples were re-adjusted to pH 8 by addition of small volumes of 1M NaOH. Fluorescence was measured as described above. The quenching of CF fluorescence at low pH clearly presented a problem for comparing real time data at different pH values. Thus, real time CF release induced by the pH independent pore forming peptide melittin appeared to be reduced at low pH but the true signal in end point supernatants were all revealed as equivalent values if adjusted to neutral pH. We therefore normalised CF release at each pH value relative to the endpoint value induced by melittin at that pH.

### DSP cross-linking

VP4His was incubated in the presence or absence of liposomes (50 µM lipid) at 25°C for 1 hr, before addition of the membrane permeable cross-linker DSP (Thermo scientific) to final concentration of 0.5 mM or 1 mM and incubation for a further 20 min at 25°. The reaction was terminated by the addition of 1 M Tris pH 7.5. Samples were analysed by SDS-PAGE under non-reducing conditions. If required, cross-linking was reversed by the addition of 50 mM DTT and incubation at 37°C for 30 min., before analysis by SDS-PAGE.

### Polyacrylamide gel electrophoresis (PAGE) and western blot

For native PAGE, purified VP4His (5 µg) was vacuum dried and rehydrated by shaking at 25°C in either liposomes (equivalent to 50 µM lipid) in 107 mM NaCl, 10 mM HEPES pH 7.4, or 300 mM diheptanoyl-*sn*-glycero-3-phosphocholine (DHPC) in 20 mM HEPES pH 7.4. Samples were mixed with an equal volume of 30% glycerol, 0.05% bromophenol blue, 150 mM Tris-HCl pH 7.0, prior to native PAGE using a 4 to 20% gradient gel (TGX, Bio-Rad). Samples were not heated prior to electrophoresis. Protein was visualised by silver staining (Pierce). For SDS-PAGE, samples were heated at 96°C for 10 min in 10% glycerol, 2% (w/v) SDS, 0.01% (w/v) phenol red, 62.5 mM Tris-HCl pH 6.8 (@ 25°C). Proteins were separated using 12% or 16.5% polyacrylamide gels and detected by staining with Coomassie brilliant blue R-250 (Sigma) or by silver staining (Pierce). Alternatively, proteins were transferred to polyvinyl difluoride (PVDF) membrane and identified using anti-VP4 sera (raised against a peptide corresponding to the C-terminus of VP4), a peroxidase-conjugated secondary antibody (Sigma) and ECL substrate (Thermo scientific).

### Electron microscopy (EM)

EM grids bearing a carbon support were glow discharged for 15 s using an EMS 100× (EMS). VP4GST was reconstituted in DPC micelles by rehydrating in 100 mM DPC, 150 mM NaCl, 20 mM sodium acetate pH 5.5 and micelles containing VP4GST were purified by size exclusion chromatography (Superose 6 1/300, GE Healthcare), adsorbed onto grids and stained using 0.75% uranyl formate. Images in Fig. 6A were collected on a JEOL 1200EX operating at 80 kV. For large image data sets, zero-loss filtered images were collected on a Zeiss Cs-TEM MC Libra200 operating at 80 kV equipped with an in-column filter set to 10 eV, and an Ultrascan 4000 CCD camera (Gatan).

### Image processing

Particles were picked with a semi-automated algorithm implemented in EMAN2 [53], e2boxer.py. All further processing was done in SPARX [54]. The particles were aligned, and classified using a K-means algorithm, which produced 7 classes. These classes were then used as references in a multi-reference alignment. To obtain an estimate of the rotation symmetry present in each of the classes, each class average was rotated and cross-correlated with the original for all angles ranging from 0 to 359 degrees in increments of 1 degree. The cross-correlation coefficient was then plotted versus the rotation angle, and number of peaks and peak separation inspected for likely n-fold symmetry.

## Supporting Information

Figure S1 **Migration of VP4His is not altered when diluted in a constant concentration of DMSO.** The indicated amounts of VP4His were diluted in a standard volume of DMSO, subjected to SDS-PAGE and visualized by silver staining. Molecular mass markers (in kilodaltons) are indicated on the left.
(TIF)

Figure S2 **Carboxyfluorescein (CF) fluorescence is quenched by low pH and detergent.** **A**. CF-containing samples were adjusted to pH 5.5, 6.5 or 7.0 and fluorescence recorded, samples were re-adjusted to pH 8 (‘recovered’) and fluorescence recorded again. **B**. CF released from liposomes by the pH dependent peptide GALA (1 µM) or the pH independent peptide melittin (10 µM), or by 0.5% (v/v) detergent TX-100.
(TIF)

Figure S3 **Micrograph image of DPC micelles in the absence of VP4.** Prepared as described in materials and methods except samples were not subjected to size exclusion chromatography. The difference in appearance and contrast in this figure (relative to figure 6) may therefore be due to a higher sample concentration. Scale bar = 20 nm.
(TIF)
