# Supplementary figures and images for: Capsid Protein VP4 of Human Rhinovirus Induces Membrane Permeability by the Formation of a Size-Selective Multimeric Pore
Source: PLoS Pathog. 2014 Aug 7;10(8):e1004294. doi: 10.1371/journal.ppat.1004294 (PMC4125281; doi:10.1371/journal.ppat.1004294)

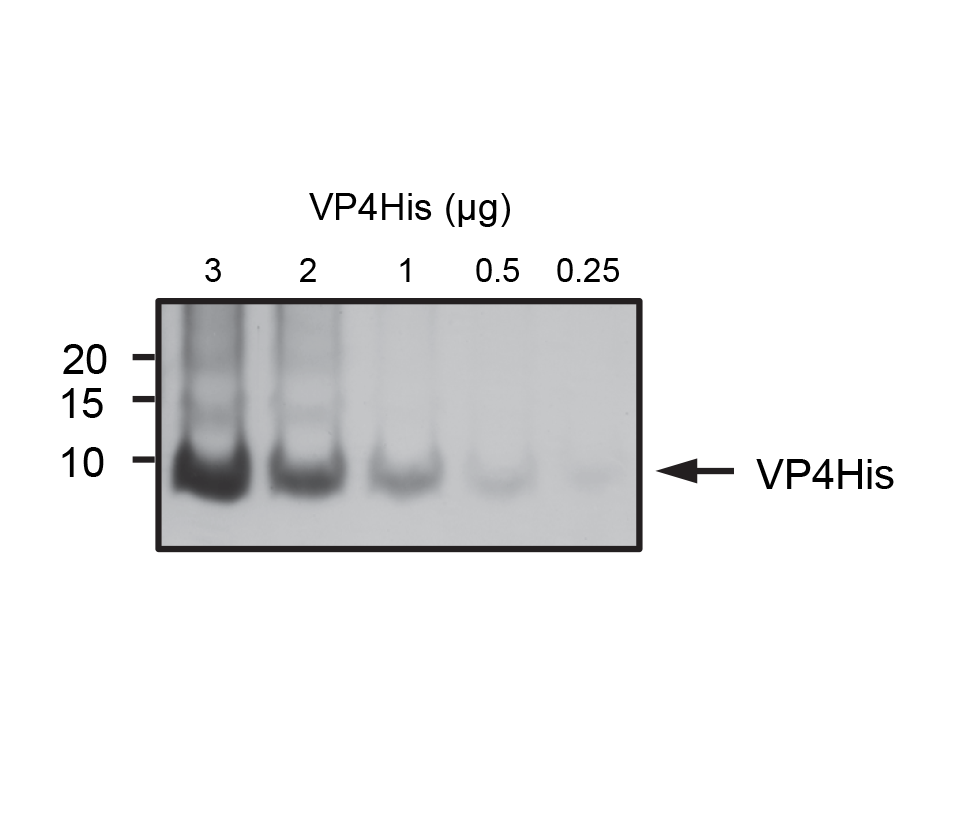

Supplement: Figure S1 — Migration of VP4His is not altered when diluted in a constant concentration of DMSO. The indicated amounts of VP4His were diluted in a standard volume of DMSO, subjected to SDS-PAGE and visualized by silver staining. Molecular mass markers (in kilodaltons) are indicated on the left. (TIF) [file ppat.1004294.s001.tif]

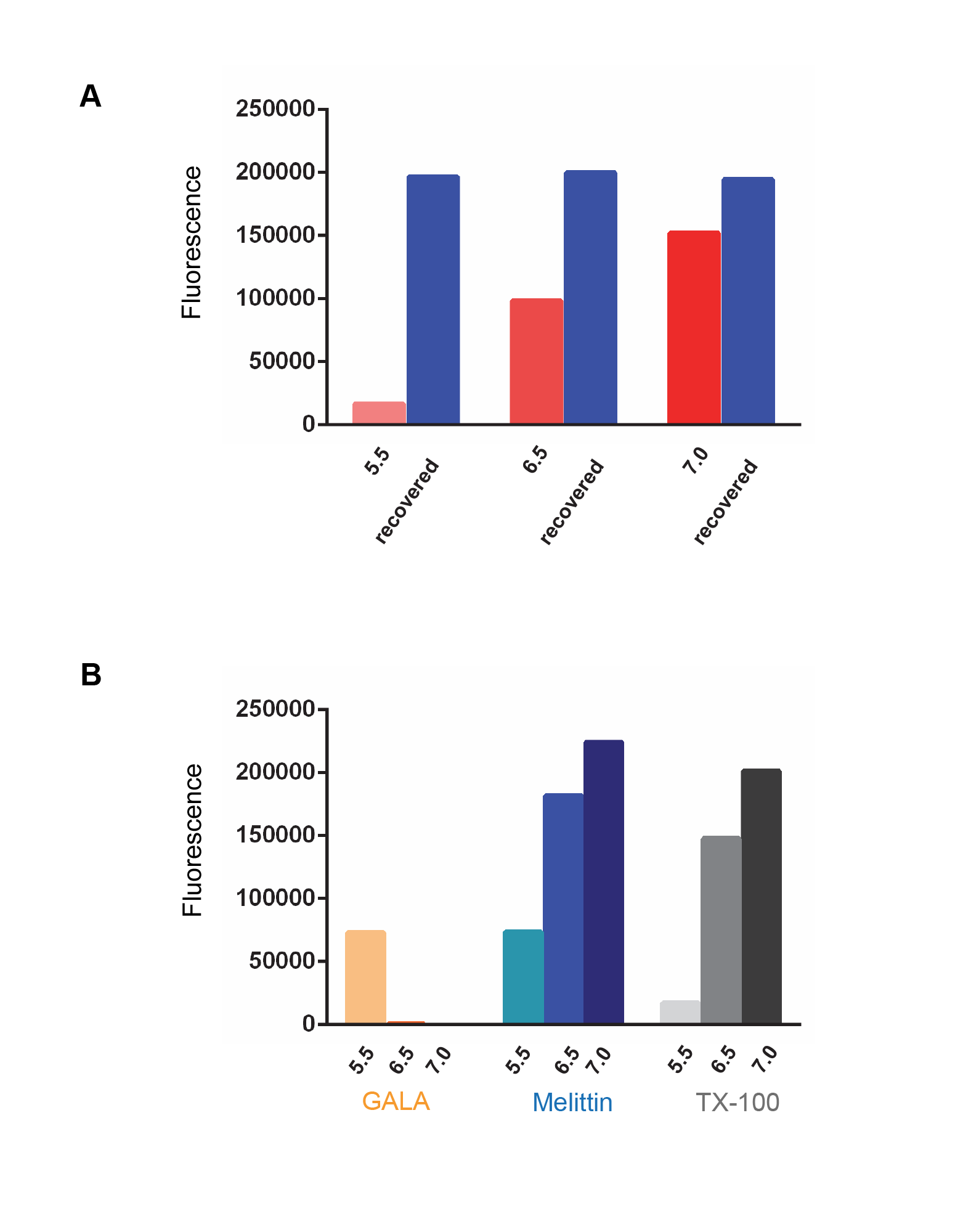

Supplement: Figure S2 — Carboxyfluorescein (CF) fluorescence is quenched by low pH and detergent. A. CF-containing samples were adjusted to pH 5.5, 6.5 or 7.0 and fluorescence recorded, samples were re-adjusted to pH 8 (‘recovered’) and fluorescence recorded again. B. CF released from liposomes by the pH dependent peptide GALA (1 µM) or the pH independent peptide melittin (10 µM), or by 0.5% (v/v) detergent TX-100. (TIF) [file ppat.1004294.s002.tif]

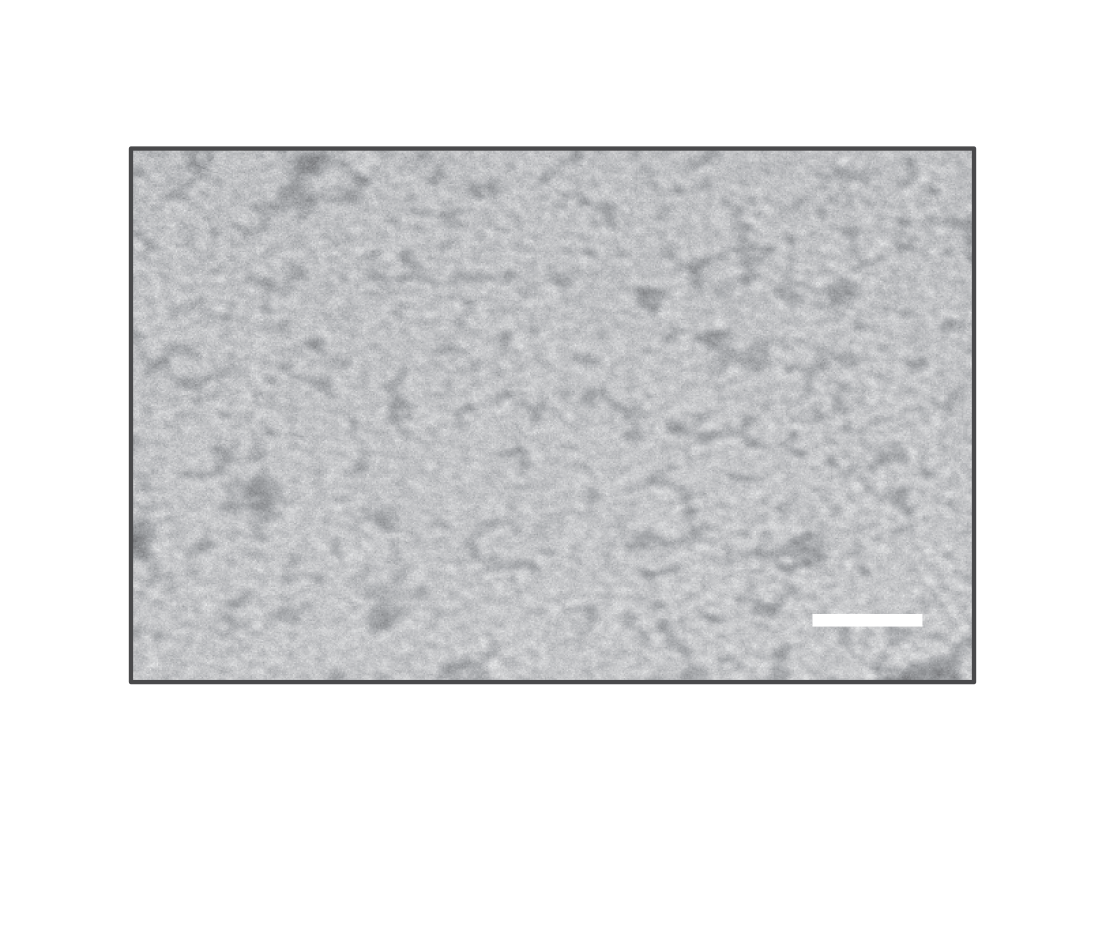

Supplement: Figure S3 — Micrograph image of DPC micelles in the absence of VP4. Prepared as described in materials and methods except samples were not subjected to size exclusion chromatography. The difference in appearance and contrast in this figure (relative to figure 6) may therefore be due to a higher sample concentration. Scale bar = 20 nm. (TIF) [file ppat.1004294.s003.tif]
